# Shaping a positive occupational safety climate in general practice teams—findings of the baseline survey of the cluster randomized IMPROVE*job* trial

**DOI:** 10.3389/fpubh.2025.1477930

**Published:** 2025-04-16

**Authors:** Anke Wagner, Esther Rind, Stephanie Burgess, Irina Böckelmann, Beatrice Thielmann, Helen Heinz, Achim Siegel, Verena Schröder, Karl-Heinz Jöckel, Anika Hüsing, Claudia Pieper, Anna-Lisa Eilerts, Tanja Seifried-Dübon, Florian Junne, Brigitte Werners, Annegret Dreher, Lukas Degen, Birgitta M. Weltermann, Monika A. Rieger

**Affiliations:** ^1^Institute of Occupational and Social Medicine and Health Services Research, University Hospital Tübingen, Tübingen, Germany; ^2^Faculty of Medicine, Institute of Occupational Medicine, Otto von Guericke University Magdeburg, Magdeburg, Germany; ^3^Center for Clinical Trials, University Hospital Essen, University of Duisburg-Essen, Essen, Germany; ^4^Institute for Medical Informatics, Biometry and Epidemiology, University Hospital Essen, University of Duisburg-Essen, Essen, Germany; ^5^Department of Psychosomatic Medicine and Psychotherapy, University Hospital Tübingen, Tübingen, Germany; ^6^Operations Research, Institute of Management, Ruhr University Bochum, Bochum, Germany; ^7^Institute of General Practice and Family Medicine, University Hospital Bonn, Bonn, Germany

**Keywords:** occupational health, occupational safety climate, working conditions, general practice, physicians, primary care, regression analysis, Germany

## Abstract

**Introduction:**

The consideration of occupational health and safety can support the creation of good sustainable working conditions in general practices and help in retaining staff and support their workability. This study aimed to assess attitudes of primary care physicians and practice assistants toward occupational safety climate, and to identify what factors are associated with a perceived positive occupational safety climate in this setting. The identification of such factors in general practice settings can serve as a basis for further developments of specific tailored interventions and offers to promote workplace safety for GPs and practice assistants.

**Methods:**

This study is based on baseline data of a cluster randomized controlled trial (IMPROVE*job* study): 84 practice owners, 28 employed physicians, and 254 practice assistants from 60 German general practices took part in a standardized survey. Occupational safety climate was measured with items from previous studies. Standardized and validated items regarding working conditions, work behavior, general health, burnout and chronic stress were also included. All statistical analyses were performed with IBM SPSS version 28, and comprised descriptive analyses, Mann–Whitney-*U*-test and Kruskal-Wallis test, as well as a stepwise multiple regression analysis considering cluster effects.

**Results:**

We found a positive perception of occupational safety climate across all occupational groups, for example regarding the role of the direct supervisor in occupational safety at work or the occupational safety commitment of the practice. Bivariate analysis mainly revealed associations between occupational safety climate and several aspects of working conditions. The regression model revealed the following important factors for perceived positive occupational safety climate (assessed by the scale company standards): supervisor support for occupational safety (β = 0.43) and job satisfaction (β = 0.22).

**Discussion:**

Leadership and job satisfaction were identified as main factors shaping a positive occupational safety climate (scale company standards) in our regression model built on data from German general practices and their practice teams. The findings are consistent with a previous study conducted in the German healthcare setting. The promotion of these factors should be supported further and can probably contribute to improving the occupational safety climate in general practices in Germany.

## Introduction

Occupational safety climate as a relevant concept can be understood as follows: “(…) the overall shared perception that a work environment in a healthcare organization is free from harm or danger under usual conditions. It consists of the explicit characteristic of safety culture in a healthcare organization influencing employee practices and attitudes toward work safety, and it thus influences occupational safety and the quality of patient care” (1). So far, the terms safety climate and safety culture have often been used synonymously due to their close relationship, and there is an ongoing discussion as to whether both terms should be considered equal or not (2). The wording “safety culture” was first mentioned after the Chernobyl nuclear power disaster in 1988, and the concept “safety climate” was already introduced by Zohar in 1980 for industrial workplaces (3). Safety climate is according to Wiegmann et al. (4) “(…) the temporal state measure of safety culture, subject to commonalities among individual perceptions of the organization. It is therefore situationally based, refers to the perceived state of safety at a particular place at a particular time, is relatively unstable, and subject to change depending on the features of the current environment or prevailing conditions.” In different workplaces, several studies were conducted so far to investigate safety climate or psychosocial safety climate and their links to working conditions and other factors [see for example (5–12)]. Further research on antecedents of a safety climate revealed for example the following seven variables as important: structural attributes of the work environment, symbolic social interaction, group and organization leadership, psychological work ownership, organizational commitment, job stress, burnout, and personality (13). He et al. (14) identified in their quantitative review situational factors (e.g., job and organizational characteristics, leadership, co-worker influence), interpersonal interactions (e.g., leader-member exchange, team-member exchange), and personal factors (e.g., personality characteristics, demographics) as pivotal requirements for a safety climate.

In 1999, the landmark report “To err is human” of the Institute of Medicine (IOM) showed for the healthcare sector the importance of a good safety climate for patients and employees all over the world (15). As a consequence, various research and actions on safety climate in the healthcare setting followed. Though, the majority of studies so far have investigated safety climate mainly in hospitals and have focused more on patient safety [see for example (16–23)] than on occupational safety for healthcare providers (1, 24). Thus, more research on occupational safety and on occupational safety climate in the primary care setting is urgently needed.

Occupational safety climate in general practices is particularly relevant because, as small, highly integrated working environments, they are exposed to unique challenges and stressors that have a direct impact on employee health and the quality of patient care (25). Currently, in different countries and especially in Germany main challenges for this specific setting are for example healthcare worker shortages, demographic change, multimorbidity of patients, and increasing and changing demands on the quality of patient care (26, 27). Working conditions in general practices are often characterized by high perceived chronic stress due to increased workload, high administrative and bureaucratic burden, increased demands and expectations from patients, lack of support from colleagues, lack of recognition from society, insufficient time, and long working hours (28–30). In fact, general practices are unique work environments in which general practitioners (GPs) act as entrepreneurs of small or micro businesses, and holding at the same time multiple roles such as employer, leader, physician and member of an interdisciplinary team (31).

The improvement of working conditions, the design of workplace safety in this challenging working environment, and the consideration of structural and behavioral prevention for personnel in general practices are fundamental. In order to effectively address the challenges in general practices and sustainably improve working conditions, both structural prevention, which involves structural changes in the working environment and working conditions, and behavioral prevention, which focuses on individual behavioral changes, are crucial. Sustainable strategies are needed to retain staff and keep them healthy and are for example demanded by the expert report in 2024 from the Advisory Council on the Assessment of Developments in the Health Care System and in Nursing Care in Germany (27). The topic workplace safety among general practice staff and its implications for behavioral and structural prevention has hardly been investigated so far. A previous study explored the need for occupational health services in primary care, and found out that expressed needs for advice and guidance on a range of occupational health issues were rarely met (32). Another study also investigated the extent of knowledge and good practice of occupational health issues for staff working in general practices in England, and found in this regard, a major need for improvement (33).

In Germany, the large collaborative IMPROVE*job* research project was conducted between 2017 and 2021 (https://www.improvejob.de/en/). This research project focused on behavioral and structural prevention to reduce psychological stress and strain in general practice teams and addressed especially working conditions, leadership and workplace safety. The project was conducted by experts, and partners from the fields in general practice and family medicine, occupational health and psychosomatic medicine, operations research, health promotion and epidemiology, and included the following four sub-projects: (1) Analysis of working conditions in primary care practices (31, 34, 35); (2) development of the multimodal participatory intervention and feasibility study; (3) evaluation of the effectiveness of the intervention in general practices by means of a cluster-randomized controlled trial (cRCT) (Registration Number: DRKS00012677) (IMPROVE*job* trial) (36–43); and (4) assessment of whether and how results can be transferred to other work environments (44). The cRCT in sub-project 3 comprised two surveys (baseline and follow-up) targeting GPs and practice assistants, thus evaluating the effects of the designed multimodal participatory intervention. The multimodal participatory intervention aimed to improve job satisfaction (primary outcome) and several secondary outcomes, among others occupational safety climate in GPs and practice assistants (36, 37). Details of the IMPROVE*job* trial and its multimodal complex intervention are published in the study protocol (36). The multimodal participatory intervention comprised two leadership workshops, a toolbox with Supplementary material, and an implementation phase of 9 months (36, 37). The IMPROVE*job* research project is an example for organizational health services research since general practices as a healthcare organization provide healthcare services to patients with diverse needs and interact with different stakeholders and changing environments at the macro, meso and micro level (45, 46).

The study reported here is based on baseline data of the IMPROVE*job* trial collected in 2019 and January 2020 before the randomization and the IMPROVE*job* intervention commenced (37). We purposely focused on baseline data of the IMPROVE*job* trial and not on the follow-up data, since only at baseline occupational safety climate was measured as comprehensively as possible. The follow-up survey comprised fewer questions on occupational safety climate.

Within this analysis, we aim to assess attitudes of GPs and practice assistants toward occupational safety climate. Furthermore, we want to exploratively identify main factors for a perceived positive occupational safety climate in this setting. The identification of relevant factors in the general practice setting can serve as a basis for further developments of specific tailored interventions and offers to promote workplace safety for GPs and practice assistants. By reporting the study, we followed the guidelines in the STROBE statement (47) (see Supplementary material 1). An additional comparison of baseline and available follow-up data on occupational safety climate stratified by intervention and control group and by the occupational groups is presented in Supplementary material 2.

## Materials and methods

### Study design, recruitment, and data collection

In this analysis, we focused on cross-sectional data of GPs and practice assistants of the cRCT called IMPROVE*job* trial. Recruitment for the cRCT started in August 2019, and study invitations were sent by letters, fax or e-mails to general practices in the North Rhine region, Germany (37). According to prior study size calculations [see for details (36)], the study team aimed to include at least 56 practices with an average of four participants per practice (37). During the recruitment process for the cRCT, 1.141 general practices were contacted per phone by researchers from the Institute for General Practice and Family Medicine, University of Bonn (37). The eligibility criteria for the cRCT were the practice owner's registration as a general practitioner in the Association of Statutory Health Insurance Physicians of North Rhine (original German: Kassenärztliche Vereinigung Nordrhein), and informed consent for the study participation of the practice owner and at least one practice assistant (37). 60 general practices agreed to participate resulting in a response rate of 5.3% (37). No interest, no time and no need with regard to an intervention aiming to reduce psychosocial stress and strain were the most indicated reasons for non-participation as revealed by a non-responder analysis (37). The data collection started in September 2019, and ended in January 2020 before the COVID-19 pandemic affected Germany. More information on the recruitment process and the data collection is described in Degen et al. (37).

### Ethics

The study was approved by the Ethics' Committee of the Medical Faculty of the University of Bonn (Reference number: 057/19), the Ethics' Committees of the Medical Association North Rhine (Lfd-Nr. 2019107) and the Ethics' Committee of medical faculty and university hospital of the University of Tübingen (Project-No.: 446/2019BO2). All participants obtained in advance written information about the study, and agreed to a written informed consent sheet which was stored at the Institute for General Practice and Family Medicine, University of Bonn.

### Questionnaire

The IMPROVE*job* trial applied for the baseline survey a detailed questionnaire covering the topics job satisfaction, working conditions, leadership, general health, work behavior, occupational safety climate, perceived chronic stress, stress coping strategies, work organizational issues as well as team activities and roles (36). The questionnaire was addressed toward practice owners, employed physicians, and practice assistants (36). A detailed overview of the whole questionnaire is described in the study protocol (36). The study presented here focused on items, indices and scales assessing occupational safety climate from the baseline questionnaire. For the correlation and regression analysis, we also included results derived at baseline by applying scales and single items regarding the topics working conditions, work behavior, general health, burnout, and perceived chronic stress as published elsewhere (37, 42, 43).

### Occupational safety climate

The questionnaire for the assessment of occupational safety climate was mainly based on previous studies (48–51). Although there are already some well-established measurement tools for safety climate in the primary care and the healthcare setting (52–54), we focused in our study on particular variables that represent the core of occupational safety climate and the specific situation in German general practices. For the baseline survey, we therefore included the following items, indices and scales:

1) one single item from the evaluation of the Joint German Occupational Safety and Health Strategy [Gemeinsame Deutsche Arbeitsschutzstrategie (GDA)] addressing evaluation of information on perceived hazards and health risks at work (How well informed do you feel in general about all the hazards and health risks associated with your work?) on a Likert scale from 1 (=very good) to 5 (=poor) (51);
2) an index regarding the subjective assessment of specific protective measures related to work-related infectious diseases (e.g., protective gloves). The index was employed previously in the WorkSafeMed study for hospital staff (48, 49), then slightly modified for the target group in the IMPROVE*job* trial and consisted of seven items regarding protective gloves, protective gowns, respiratory protection, containers for dropping needles, hygiene instructions, hand and surface disinfection and clearly regulated procedure after a needlestick injury. Cronbach's alpha was computed to determine the internal consistency reliability (Cronbach's α = 0.67). The items were answered on a Likert scale from 1 (strongly agree) to 5 (strongly disagree). Low values indicate a more positive perception of occupational safety climate;
3) an index regarding the personal perception of the frequency of occupational risks previously employed in the WorkSafeMed study (Do you feel exposed to risks of infection?) (48, 49) (Cronbach's α = 0.66). Four items were answered on a 5-point Likert scale of frequency (from 1 = always to 5 = never). Here, low values on single items imply a rather negative perception of occupational safety climate;
4) a modified version with nine items of the scale company standards (Betriebliche Normen) of the FAGS questionnaire [Fragebogen zum Arbeits- und Gesundheitsschutz (Questionnaire on Occupational Safety and Health)] on the priority of occupational health and safety in the workplace and the extent to which employees identify with these standards (50, 55, 56). As this scale, in contrast to the other occupational safety climate variables in the questionnaire, refers to the overarching context of health and occupational safety in practice (see single items in Supplementary material 3), we regard it as a good indicator of occupational safety climate, and used it as main outcome variable in the regression analysis (Cronbach's α = 0.89). Items were rated on a 5-point Likert scale from 0 (=strongly disagree or never) to 4 (=strongly agree or always), and here high values indicate a rather positive perception of occupational safety climate. Five negatively worded items were recoded before scale calculation as recommended (50); and
5) a single item also from the evaluation of the Joint German Occupational Safety and Health Strategy assessing the occupational safety commitment of the practice (e.g., Overall, how would you rate your practice's occupational health and safety commitment?) (51). This item was also rated on a Likert scale reaching from 1 (=very high) to 4 (=very low).

The questionnaire for practice owners differed slightly from the questionnaire for employed physicians and practice assistants and included further questions from the Joint German Occupational Safety and Health Strategy (51). Thus, the questionnaire for practice owners included six additional items to assess the level of knowledge of legal occupational health and safety regulations, which were answered on a Likert scale from 0 (=very low) to 3 (=very high) (51). In addition, the practice owners were asked to rate the value of occupational risk assessments for the promotion of occupational safety by a single item with the following four answer options: 1 (=very high), 2 (=rather high), 3 (=rather low), and 4 (=very low) (51).

The questionnaire for employed physicians and practice assistants included three additional single items regarding the behavior of the direct supervisor: “…openly addresses problems concerning occupational safety in our practice”/“…focuses more on occupational safety than a year ago”/“It is important to my direct supervisor that our practice pays great attention to occupational safety”. Each item was answered on a 5-point Likert scale from 1 (=strongly disagree) to 5 (=strongly agree). Also, the scale supervisor support for occupational safety of the WorkSafeMed study (48, 49) was applied with three items reaching from 1 (=strongly disagree) to 5 (=strongly agree) (Cronbach's α = 0.80). Here, one negatively coded item was recoded before scale calculation.

### Working conditions

The questionnaire for working conditions involved various scales of the third version of the German Copenhagen Psychosocial Questionnaire (COPSOQ) (Version 2018) (57, 58). We used items of the following scales in the baseline questionnaire: job satisfaction (Cronbach's α = 0.83), quantitative demands (Cronbach's α = 0.68), emotional demands (Cronbach's α = 0.68), work pace (Cronbach's α = 0.50), work-privacy conflict (Cronbach's α = 0.88), delimitation (Cronbach's α = 0.22), predictability (Cronbach's α = 0.81), role clarity (Cronbach's α = 0.77), role conflicts (Cronbach's α = 0.77), social support (Cronbach's α = 0.80), feedback (Cronbach's α = 0.49), social relations (single item), bullying (single item), and sense of community (Cronbach's α = 0.85). Items of the scales were transformed as recommended, ranging from 0 (=lowest value, do not agree at all) to 100 (=highest value, totally agree) (57, 58). Depending on the scales, a higher value can be interpreted as positive (e.g., for job satisfaction, predictability, role clarity, social support, feedback, social relations, and sense of community) or negative (e.g., for quantitative demands, emotional demands, work pace, work-privacy conflict, delimitation, role conflicts, and bullying).

### Work behavior

For capturing work behavior, we applied the short version of the Occupational Self-Efficacy Scale (BSW) (59), and the short version of the Work-related Behavior and Experience Patterns questionnaire (AVEM-44, Arbeitsbezogenes Verhaltens- und Erlebnismuster) (60). The short version of the BSW comprised eight items on a six-point Likert scale ranging from 1 (=completely true) to 6 (=not at all true) (59) (Cronbach's α = 0.90). The eight items are summed up in a total score (59).

The AVEM-44 included 44 items and identified 11 work-related behavior and experience dimensions from the following three areas (61):

- engagement with work (dimensions: subjective importance of work, work-related ambition, willingness to work until exhausted, striving for perfection, and distancing ability) (each score Cronbach's α ≥ 0.71),
- resilience in dealing with the everyday stress of work (dimensions: distancing ability, tendency to resignation in the face of failure, proactive problem-solving, and inner calm and balance) (each score Cronbach's α ≥ 0.71), and
- emotions associated with work and life in general (dimensions: experience of success at work, satisfaction with life, and experience of social support) (each score Cronbach's α ≥ 0.75).

Each dimension comprised four items, which are measured on a 5-point Likert scale ranging from 1 (=not at all) to 5 (=completely) (61). Distancing ability is an important component of the first two areas (engagement with work and resilience to stress) (61). The AVEM dimensions were converted into stanine scores ranging from 1 to 9. The normal range for each dimension is between 4 and 6 stanine scores. AVEM identifies four patterns which describe coping strategies for occupational stress: healthy ambitious (pattern G), unambitious (pattern S), excessively ambitious (risk pattern A), and burnout (risk pattern B) (61). Considering the manual, all subjects were classified into risk patterns (A, B) or health-promoting patterns (G, S) based on the levels of expression for all dimensions (60).

### General health

General health was measured with the World Health Organization-Five Well-Being Index (WHO-5) (62). The WHO-5 comprised five items asking for the frequency of certain feelings in the last two weeks with a six-point Likert scale from 5 = all of the time to 0 = at no time (62). The values of the items are added to a sum-score from 0 to 25 and are then multiplied by 4 to present the final score (62) (Cronbach's α = 0.89). The final score ranged from 0 = denoting the worst and 100 = showing the best perceived wellbeing (62).

### Burnout and perceived chronic stress

Burnout was assessed with the following two items from the Maslach Burnout Inventory (63): emotional exhaustion: “I feel burned out from my work”, and depersonalization: “I have become more callous toward people since I took this job”. Within the IMPROVE*job* trial, for each item, the response options were on a 6-point Likert scale ranging from 0 (=never) to 5 (=very often). Thus, high values imply a higher likelihood or expression for emotional exhaustion and depersonalization (64).

Perceived chronic stress was assessed with the German short version of the Screening Scale of the Trier Inventory for the Assessment of Chronic Stress (TICS-SSCS) (65, 66). This instrument consisted of 12 items rated on 5-point Likert scales from 0 (never) to 4 (very often), and measured retrospectively strain due to perceived chronic stress for the last 3 months. The values of the TICS-SSCS are added to a sum-score ranging from 0 to 48 with 0 meaning never stressed and 48 meaning very often stressed (Cronbach's α = 0.91) (66).

### Statistical analysis

Statistical analysis was performed using IBM SPSS version 28 for Windows (IBM Corp., Armonk, NY, USA). Since there were very few variables in the baseline questionnaire with missing values [all variables <5% missing values with exception of the scale experience of social support (*n* = 48, 13.1%)], they were not imputed. We conducted descriptive analyses with mean values, standard deviations, median values and the range of continuous variables and scale-scores. The aim was to ensure the comparison of scales/indices and individual items. We tested the metric variables for normal distribution using the Shapiro-Wilk-test. Since the requirements for a parametric test were not fulfilled, we applied the Kruskal-Wallis *H* test or the Mann-Whitney-*U*-test for the detection of differences regarding attitudes of the three occupational groups toward occupational safety climate. We thereby considered a potential cluster effect from sampling practices by adjusting the standard deviations for each occupational group. A *p*-value <0.05 was considered as statistically significant. By using the Kruskal-Wallis *H* test, the *post-hoc* Bonferroni correction was applied, when we identified statistically significant differences between the occupational groups. We calculated and categorized the effect size according to Cohen's suggestions: d_Cohen_ <0.30 = small effect/difference, d_Cohen_ <0.50 = medium effect/difference and d_Cohen_ ≥ 0.50 = large effect/difference (67). The statistical analysis further comprised Spearman's correlation analyses, and a stepwise regression analysis using the scale company standards as dependent variable since it served as a rather good indicator for perceived occupational safety climate. We considered potential cluster effects by using Generalized Estimating Equations (GEE) (68, 69). By using the Spearman's correlation coefficients (rho), we controlled for multiple testing by computing Bonferroni corrected *p*-values (70). Due to the many statistical tests and the variable selection procedures used, the *p*-values should be viewed with caution. It should be emphasized that all *p*-values in this paper are to be interpreted strictly descriptively.

## Results

### Sample characteristics

84 practice owners, 28 employed physicians and 254 practice assistants of 60 general practices took part in the baseline survey (total sample of 366 participants). The general practices were in the outpatient setting, and included 21 solo-practices, and 39 group-practices (37). As typical for German general practices, the involved practice owners fulfilled at the same time multiple roles such as entrepreneur, GP, and employer for the employed physicians and practice staff. The practice assistants were trained as medical assistants, but some of them had further qualifications (e.g., wound management, diabetes, quality management). Practice owners worked predominantly full-time, whereas employed physicians worked mainly part-time. Table 1 shows the descriptive baseline characteristics of the three occupational groups. More characteristics of the sample are described in Degen et al. (37). Regarding the distribution of AVEM patterns in our sample, we described in a previous analysis of baseline data of the IMPROVE*job* trial sample the following results: 19.5% (*n* = 44) of the study participants had risk pattern B, 8.4% (*n* = 19) had risk pattern A, 23.0% (*n* = 52) had health-promoting pattern G and 49.1% (*n* = 111) had health-promoting pattern S (43). For 88 subjects, no assignment was possible, and 52 subjects showed a pattern combination (43).

**Table 1:** Sociodemographic characteristics of the participants (*N* = 366) (37).

**Variable**	**Practice owners (*n* = 84)**	**Employed physicians (*n* = 28)**	**Practice assistants (*n* = 254)**
Male (in %)	47.6%	21.4%	0.4%
Female (in %)	52.4%	78.6%	99.6%
Working full time in %	90.5%	28.6%	41.5%
Working part time in %	9.5%	71.4%	58.5%
Age in years: Mean ± SD	54.3 ± 6.2	44.8 ± 9.8	41.0 ± 13.0
Overall duration of the employment in years: Mean ± SD	15.8 ± 8.3	4.5 ± 5.5	9.4 ± 8.8
Years since accreditation: Mean ± SD (only for physicians)	26.6 ± 7.2	16.3 ± 9.7	-
Overall duration in the profession in years: Mean ± SD (only for practice assistants)	-	-	19.9 ± 13.3

### Perspectives on occupational safety climate

Overall, we detected positive attitudes toward occupational safety climate among the three occupational groups (see Table 2). We observed no statistically significant difference between the three occupational groups regarding the evaluation of information on perceived hazards and health risks at work (*p* = 0.735), the assessment of specific protective measures related to work-related infectious diseases (*p* = 0.494), and attitudes regarding company standards (*p* = 0.722). The index personal perception of the frequency of occupational risks was evaluated significantly more positively by practice assistants than by practice owners (*p* = 0.035). Furthermore, the item occupational safety commitment of the practice was rated significantly more positively by employed physicians than by practice owners (*p* = 0.025). The identified differences represented a rather small effect according to Cohen. A further comparison of study participants who worked full-time or part-time with regard to their perceptions of occupational safety climate is presented in Supplementary material 4. Here, we also considered a potential cluster effect from sampling practices by adjusting the standard deviations for each group. Our results showed that part-time workers rated their occupational safety climate more positively than full-time workers (see for example results for personal perception of the frequency of occupational risks, occupational safety commitment of the practice, and supervisor support for occupational safety).

**Table 2:** Descriptive results and differences regarding attitudes toward occupational safety climate.

**Occupational group**		**Practice owners**	**Employed physicians**	**Practice assistants**	**Significance**		**Effect size**

**Topic**	**Variables**	***n*** **Mean** ±**SD Median (Min-Max)**	***n*** **Mean** ±**SD Median (Min-Max)**	***n*** **Mean** ±**SD Median (Min-Max)**	***p***	***Post-hoc p**_*Bonferroni*_*	***d**_*Cohen*_*
Occupational safety climate (48–51, 55, 56)	Evaluation of information on perceived hazards and health risks at work^1^ (single item, 1 = positive; 5 = negative)	*n* = 81 2.0 ± 1.3 2.0 (1–4)	*n* = 28 2.0 ± 0.9 2.0 (1–4)	*n* = 244 2.0 ± 1.1 2.0 (1–5)	0.735^a^	-	0.126
	Assessment of specific protective measures related to work-related infectious diseases^2^ (index, 7 items, 1 = positive; 5 = negative)	*n* = 84 1.7 ± 0.6 1.7 (1–3)	*n* = 28 1.6 ± 0.7 1.6 (1–3.14)	*n* = 251 1.7 ± 0.6 1.7 (1–3.43)	0.494^a^	-	0.081
	Personal perception of the frequency of occupational risks^2^ (index, 4 items, 5 = positive; 1 = negative)	*n* = 84 3.9 ± 0.9 4.0 (2–5)	*n* = 28 4.0 ± 0.8 4.0 (3–5)	*n* = 253 4.0 ± 0.9 4.0 (1.5–5)	0.039^a^	Practice owners vs. practice assistants = 0.035	0.224
	Company standards^3^ (scale, 9 items, 4 = positive; 0 = negative)	*n* = 82 3.0 ± 0.7 2.9 (1.56–3.89)	*n* = 24 3.0 ± 0.7 3.0 (1.89–3.89)	*n* = 243 2.8 ± 0.9 3.0 (0.11–4)	0.722^a^	-	0.125
	Occupational safety commitment of the practice^1^ (single item, 1 = positive; 4 = negative)	*n* = 84 2.3 ± 0.6 2.0 (1–3)	*n* = 27 1.9 ± 0.7 2.0 (1–3)	*n* = 249 2.1 ± 0.7 2.0 (1–4)	0.016^a^	Practice owners vs. employed physicians = 0.025	0.268
	My direct supervisor openly addresses problems concerning occupational safety in our practice^2^ (single item, 5 = positive; 1 = negative)	-	*n* = 27 4.0 ± 1.1 4.0 (2–5)	*n* = 250 3.8 ± 1.1 4.0 (1–5)	0.545^b^	-	0.069
	My direct supervisor focuses more on occupational safety than a year ago^2^ (single item, 5 = positive; 1 = negative)	-	*n* = 21 2.7 ± 2.0 3.0 (1–5)	*n* = 237 2.7 ± 1.3 3.0 (1–5)	0.947^b^	-	0.008
	It is important to my direct supervisor that our practice pays great attention to occupational safety^2^ (single item, 5 = positive; 1 = negative)	-	*n* = 25 3.8 ± 1.0 4.0 (3–5)	*n* = 252 3.9 ± 1.1 4.0 (1–5)	0.624^b^	-	0.056
	Supervisor support for occupational safety^2^ (scale, 3 items, 5 = positive; 1 = negative)	-	*n* = 28 4.1 ± 0.9 4.3 (2–5)	*n* = 253 4.0 ± 0.9 4.3 (1–5)	0.609^b^	-	0.06

SD, Standard deviation.

^a^Kruskal-Wallis H test.

^b^Mann-Whitney-U-test.

^1^Questions from the Evaluation of the Joint German Occupational Safety and Health Strategy (51).

^2^WorkSafeMed study (48, 49).

^3^FAGS questionnaire [Fragebogen zum Arbeits- und Gesundheitsschutz (Questionnaire on Occupational Safety and Health)] (50, 55, 56).

We found a positive assessment of the role of the direct supervisor in relation to occupational safety at work, taking into account the perspectives of the practice assistants and the employed physicians. There were no statistically significant differences between these two occupational groups regarding the three respective items to evaluate the direct supervisor: “My direct supervisor openly addresses problems concerning occupational safety in our practice” (*p* = 0.545), “My direct supervisor focuses more on occupational safety than a year ago” (*p* = 0.947), and “It is important to my direct supervisor that our practice pays great attention to occupational safety” (*p* = 0.624). The scale supervisor support for occupational safety was also evaluated quite positively and revealed no statistically difference between practice assistants and employed physicians (*p* = 0.609). Table 2 presents further details on all descriptive results of the three occupational groups.

Practice owners rated their level of knowledge regarding legal occupational health and safety regulations in the medium range (Mean ± SD = 1.7 ± 0.6) [index, 3 items, range 0 (very low) to 3 (very high)]. The benefit of occupational risk assessments for the promotion of occupational safety was rated very differently. 56% of the practice owners perceived the benefit as very high or rather high whereas 44% considered the benefit as rather low or very low.

### Associations between perceived occupational safety climate and other variables

We performed bivariate analyses between the dependent variable company standards and all other variables. The highest statistically significant content-related positive associations after adjusting for multiple testing were identified for occupational safety commitment of the practice [Spearman's ρ = −0.668 (item with reverse polarity)], for supervisor support for occupational safety (Spearman's ρ = 0.633), and the two single items regarding the behavior of the direct supervisor: “It is important to my direct supervisor that our practice pays great attention to occupational safety” (Spearman's ρ = 0.545) and “My direct supervisor openly addresses problems concerning occupational safety in our practice” (Spearman's ρ = 0.447). Further positive associations were found for job satisfaction (Spearman's ρ = 0.522), predictability (Spearman's ρ = 0.407), and the WHO-5 score (Spearman's ρ = 0.357) and evaluation of information on perceived hazards and health risks at work [Spearman's ρ = −0.343 (item with reverse polarity)]. Further results are reported in Table 3.

**Table 3:** Results of the correlation analysis between company standards (dependent variable) and all other variables (strongest positive correlation coefficients for each topic of the questionnaire are shown first in the table).

**Independent variables**	**Dependent variable (company standards)**			

	**Spearman correlation coefficient**	***p*** **-values**	**Bonferroni corrected** ***p*****-values**	***N***
**Occupational safety climate**				
Occupational safety commitment of the practice (single item) [item with reverse polarity]^1^	−0.668	<0.001^**^	0.038^*^	347
Supervisor support for occupational safety (scale)^2^	0.633	<0.001^**^	0.038^*^	267
It is important to my direct supervisor that our practice pays great attention to occupational safety (single item)^2^	0.545	<0.001^**^	0.038^*^	267
My direct supervisor openly addresses problems concerning occupational safety in our practice (single item)^2^	0.447	<0.001^**^	0.038^*^	266
Evaluation of information on perceived hazards and health risks at work (single item) [item with reverse polarity]^1^	−0.343	<0.001^**^	0.038^*^	339
Personal perception of the frequency of occupational risks (index)^2^	0.342	<0.001^**^	0.038^*^	349
My direct supervisor focuses more on occupational safety than a year ago (single item)^2^	0.004	0.950	>0.999	251
Assessment of specific protective measures related to work-related infectious diseases (index)^2^	−0.184	0.001^**^	0.038^*^	347
**Working conditions**				
Job satisfaction (scale)^3^	0.522	<0.001^**^	0.038^*^	349
Predictability (scale)^3^	0.407	<0.001^**^	0.038^*^	343
Role clarity (scale)^3^	0.304	<0.001^**^	0.038^*^	345
Social support (scale)^3^	0.282	<0.001^**^	0.038^*^	346
Sense of community (scale)^3^	0.225	<0.001^**^	0.038^*^	347
Feedback (scale)^3^	0.139	0.010^**^	0.38	344
Social relations (scale)^3^	0.027	0.623	>0.999	346
Role conflicts (scale)^3^	−0.397	<0.001^**^	0.038^*^	344
Work pace (scale)^3^	−0.343	<0.001^**^	0.038^*^	349
Quantitative demands (scale)^3^	−0.317	<0.001^**^	0.038^*^	349
Work-privacy conflict (scale)^3^	−0.302	<0.001^**^	0.038^*^	347
Bullying (scale)^3^	−0.254	<0.001^**^	0.038^*^	343
Emotional demands (scale)^3^	−0.147	0.006^**^	0.228	348
Delimitation (scale)^3^	−0.091	0.090	>0.999	347
**Work behavior**				
Occupational self-efficacy scale (scale) [scale with reverse polarity]^4^	−0.211	<0.001^**^	0.038^*^	349
Distancing ability (scale)^5^	0.232	<0.001^**^	0.038^*^	349
Satisfaction with life (scale)^5^	0.228	<0.001^**^	0.038^*^	349
Subjective importance of work (scale)^5^	0.163	0.002^**^	0.076	347
Inner calm and balance (scale)^5^	0.108	0.044^*^	>0.999	349
Experience of social support (scale)^5^	0.108	0.058	>0.999	307
Proactive problem-solving (scale)^5^	0.097	0.072	>0.999	349
Experience of success at work (scale)^5^	0.093	0.082	>0.999	349
Work-related ambition (scale)^5^	0.019	0.719	>0.999	348
Tendency to resignation in the face of failure (scale)^5^	−0.176	0.001^**^	0.038^*^	349
Willingness to work until exhausted (scale)^5^	−0.131	0.014^*^	0.532	349
Striving for perfection (scale)^5^	−0.048	0.374	>0.999	349
**General health**				
World Health Organization-Five Well-Being Index (WHO-5) (score)^6^	0.357	<0.001^**^	0.038^*^	344
**Burnout and perceived chronic stress**				
I feel burned out from my work (single item)^7^	−0.358	<0.001^**^	0.038^*^	347
I have become more callous toward people since I took this job (single item)^7^	−0.092	0.086	>0.999	347
Screening Scale of the Trier Inventory for the Assessment of Chronic Stress (TICS-SSCS) (sum score)^8^	−0.355	<0.001^**^	0.038^*^	346

^1^Questions from the Evaluation of the Joint German Occupational Safety and Health Strategy (51).

^2^WorkSafeMed study (48, 49).

^3^Copenhagen Psychosocial Questionnaire (COPSOQ) (57, 58).

^4^Occupational Self-Efficacy (BSW) (59).

^5^Work-related Behavior and Experience Patterns (AVEM) (60, 61).

^6^World Health Organization-Five Well-Being Index (WHO-5) (62).

^7^Maslach Burnout Inventory (63).

^8^Screening Scale of the Trier Inventory for the Assessment of Chronic Stress (TICS-SSCS) (65, 66).

^**^Correlation is significant at the 0.001 level (two-tailed).

^*^Correlation is significant at the 0.05 level (two-tailed).

We found the highest statistically significant negative associations for role conflicts (Spearman's ρ = −0.397), work pace (Spearman's ρ = −0.343), quantitative demands (Spearman's ρ = −0.317), the single item “I feel burned out from my work” (Spearman's ρ = −0.358), and the TICS-SSCS sum score as an indicator for perceived chronic stress (Spearman's ρ = −0.355). For these variables increasing values are accompanied by a more negative rating of occupational safety climate.

### Factors associated with a positive occupational safety climate in general practices

Based on the descriptive analyses, we developed a regression model with the aim of identifying main factors for shaping a positive occupational safety climate perceived by practice owners, employed physicians and practice assistants from the thematic fields of occupational safety climate, working conditions, work behavior, general health, burnout, and perceived chronic stress. The scale company standards served as dependent variable in our regression model since it is a rather good indicator for perceived occupational safety climate (see Statistical analysis section). In order to conceptually develop the regression model, we built on our experience from a previous study in the healthcare sector (48), and included variables in the context of stress and strain as well as variables addressing leadership and occupational safety climate (48). In advance of the stepwise regression analysis, correlation analyses were additionally carried out as recommended by Field (71) between all chosen variables and the dependent variable to avoid multicollinearity (Spearman's ρ > 0.8) (see Table 3). Only the following variables that revealed statistically significant correlations with the dependent variable were included further in the explorative regression analysis:

- Occupational safety climate including respective leadership aspects: Evaluation of information on perceived hazards and health risks at work, Personal perception of the frequency of occupational risks, Occupational safety commitment of the practice, My direct supervisor openly addresses problems concerning occupational safety in our practice, It is important to my direct supervisor that our practice pays great attention to occupational safety, Supervisor support for occupational safety
- Working conditions: Job satisfaction, Quantitative demands, Work pace, Predictability, Role conflicts
- Work behavior: Occupational Self-Efficacy Scale, Subjective importance of work, Distancing ability, Satisfaction with life
- General health: WHO-5
- Burnout: I feel burned out from my work
- Perceived Chronic Stress: TICS-SSCS

By developing the regression model, we used the backward selection method to identify suitable factors (71). We checked the final regression model for multicollinearity using the Durbin-Watson statistic and VIF values (71), and considered probable cluster effects. The final regression model revealed the following prevailed variables (factors) from different parts of the IMPROVE*job* questionnaire: supervisor support for occupational safety (β = 0.43), job satisfaction (β = 0.22), predictability (β = 0.16), personal perception of the frequency of occupational risks (β = 0.12), subjective importance of work (β = 0.11), and WHO-5 (β = 0.10) (see Table 4). The single item for burnout had no significant effect in the regression model. The model achieved an explained variance of 0.58 *R*^2^ after adjusting for cluster effects (*R*^2^ = 0.59 without adjustment).

**Table 4:** Occupational safety climate model—stepwise linear regression analysis for the outcome company standards adjusted for cluster effects.

**Variables**	***b* (95% CI)**	***SE_*B*_***	**ß**	**Chi-square**
**Constant**	−1.17 (−1.80 to −0.53)	0.32		10.90
Supervisor support for occupational safety (scale)^1^	0.44 (0.35 to 0.54)	0.05	0.43	83.22
Job satisfaction (scale)^2^	0.01(0.01 to 0.02)	0.00	0.22	16.46
Predictability (scale)^2^	0.01(0.00 to 0.01)	0.00	0.16	17.13
Personal perception of the frequency of occupational risks (index)^1^	0.13 (0.03 to 0.24)	0.05	0.12	6.97
Subjective importance of work (scale)^3^	0.04 (0.01 to 0.07)	0.02	0.11	6.68
WHO-5 (score)^4^	0.00 (0.00 to 0.01)	0.00	0.10	4.77
I feel burned out from my work (single item)^5^	−0.00 (−0.06 to 0.06)	0.03	−0.00	0.00

N = 258, R^2^ = 0.59, Adj. R^2^ = 0.58; Dependent variable: company standards (scale), Adjustment for cluster effects via Generalized Estimating Equations (GEE), CI, confidence interval.

^1^WorkSafeMed study (48, 49).

^2^Copenhagen Psychosocial Questionnaire (COPSOQ) (57, 58).

^3^Work-related Behavior and Experience Patterns (AVEM) (60, 61).

^4^World Health Organization-Five Well-Being Index (WHO-5) (62).

^5^Maslach Burnout Inventory (63).

## Discussion

We conducted an in-depth analysis on perceived occupational safety climate by using baseline data from the IMPROVE*job* trial with practice owners, employed physicians and practice assistants in general practices in Germany. To our knowledge, this is the first research focusing on perceived occupational safety climate in this very important healthcare setting.

To assess perceived occupational safety climate, we used the scale company standards as outcome, i.e., dependent variable, in our regression model. This scale was taken from the FAGS questionnaire [Fragebogen zum Arbeits- und Gesundheitsschutz (Questionnaire on Occupational Safety and Health)] on the priority of occupational health and safety in the workplace and the extent to which employees identify with these standards (50, 55, 56). The findings on occupational safety climate suggest possible implications for structural and behavioral prevention in general practices, which we discuss in the following.

### Perspectives on occupational safety climate according to occupational groups

Occupational safety climate was rated positively in general by practice owners, employed physicians and practice assistants. This may be due to a positive selection of general practices. We cannot exclude the possibility that mostly general practices participated in this trial, that considered the topics of the multimodal participatory IMPROVE*job* intervention focusing on the prevention of work-related psychosocial stress and strain and thus good working conditions as being very important.

By comparing the perspectives on occupational safety climate, we found only few differences between the occupational groups. Practice owners rated the personal perception of the frequency of occupational risks more negatively than practice assistants. They may be more aware or potentially more affected by infections, skin diseases, consequences of long working hours or even exposure to hazardous substances. This result is not surprising. Physicians are normally more involved in invasive tasks (e.g., stabbing and cutting injuries) than other occupational groups in healthcare settings, and therefore for example needlestick injuries are more often reported for physicians than for nurses (72). However, due to the high workload of GPs, the delegation of medical activities to practice assistants is discussed more frequently (73). It therefore remains important for all occupational groups to adhere to occupational health and safety regulations in the workplace. Working long hours and being affected by a higher work-privacy-conflict is also well-known for German physicians (49). In a previous analysis of baseline data of the IMPROVE*job* trial, Göbel et al. (39) found out that being a practice owner and working full-time was associated with a stronger expression of work-privacy-conflict. Another German study also identified an association between a higher perceived work-privacy-conflict and working full-time among German physicians (74). Measures to reduce long working hours as one key feature of physicians' working conditions should therefore be further implemented especially for practice owners. The comparison of study participations working full-time or part-time showed that part-time workers rated their occupational safety climate more positively than full-time workers (for further results see Supplementary material 4). This result is not surprising, since working conditions are closely linked to occupational safety climate, and full-time workers are therefore more exposed to various workplace demands. In addition, work content (e.g., specific examinations, specific group of patients) might be slightly different for full-time compared to part-time workers in general practices. Careful and reflective consideration of occupational safety climate and working conditions, also in relation to working hours and aspects of work organization, is therefore of great importance.

The item occupational safety commitment of the practice was rated significantly more positively by employed physicians than by practice owners. This suggests, that practice owners may see the need for more action in terms of occupational health and safety measures. Practice owners also rated their level of knowledge regarding legal occupational health and safety regulations in the medium range and not quite high. So, we assume that this group saw areas of improvement for occupational safety here, and was therefore motivated to take part in the IMPROVE*job* trial. Practice owners also perceived the benefit of occupational risk assessments for the promotion of occupational safety very differently: 56% of the practice owners perceived the benefit as very high or rather high whereas 44% considered the benefit only as rather low or very low. This result is different from the findings of 4.794 companies from different economic branches in the final report of the Joint German Occupational Safety and Health Strategy of the years 2008–2012 (51). In the report, 68% rated the benefit of an occupational risk assessment as rather high or very high, and 31% as rather low or very low (51). Thus, it appears, that for our sample of practice owners, the practical benefit of the legally required occupational risk assessment for the promotion of occupational safety for employers and employees should be better communicated. In the IMPROVE*job* trial, practice owners received for example some practical support and advice on how to implement the legally required occupational risk assessment in their practice as part of the multimodal complex intervention.

We also found no differences between practice assistants and employed physicians when evaluating their direct supervisor and their level of involvement with regard to occupational safety. Direct supervisors received a quite positive assessment in this regard. This positive finding also may be due to the selection of the participating practices in the IMPROVE*job* trial.

### Associations between perceived occupational safety climate and other variables

The highest content-related positive associations were found for the single item occupational safety commitment of the practice and the scale supervisor support for occupational safety as well as the two single items regarding the behavior of the direct supervisor. This result was also discovered in the WorkSafeMed study performed in two German university hospitals (48). Positive experienced leadership style and behavior seemed to have an influence on a favorable occupational safety climate (48). A recent study in hospitals in Iran investigated the association between a hospital safety and health management system, hospital safety climate and the prevalence of needlestick and sharp injuries among nurses (75). According to the authors, a good hospital safety and health management system as well as perceived positive management support for occupational safety played a crucial role in reducing the incidence of injuries in workplaces for nurses (75). Thus, leadership appeared to be an important element for creating a positive occupational safety climate.

Further positive associations were identified for job satisfaction, predictability, several aspects of work behavior, and general health. The relationship between job satisfaction and organizational practices (e.g., safety climate) was also explored in another study with hospital nurses (76). The more positively organizational practices were rated, the higher was the level of job satisfaction among the employees (76). Katz et al. (77) examined associations between workplace health and safety climate and different employee outcomes in three medium-sized manufacturing companies. The authors found associations between a strong safety climate and a favorable perceived general health (77). Thus, improving perceived occupational safety climate by respective measures could be a suitable measure to foster the perceived general health of employees. The results are not from the healthcare setting, but we believe they are applicable to our study population.

We identified rather negative associations between our dependent variable for perceived occupational safety climate and quantitative demands, work pace, role conflicts, burnout and perceived stress. Thus, poorer psychosocial working conditions characterized by high demands and increased risk for burnout and perceived psychological stress, can lead to a more negative perception of occupational safety climate. Previous studies in the healthcare setting also found associations for example between burnout and a lower perceived safety climate in the workplace (78, 79).

Overall, our results regarding the associations between occupational safety climate and working conditions in the general practice setting support our previous findings in the hospital setting (48). Improving working conditions and management support for workplace safety, as well as measures to enhance outcomes for employees (e.g., job satisfaction, general health) can lead to a more positive perception of occupational safety climate in the workplace.

### Factors associated with a positive occupational safety climate in general practices

Based on the bivariate analysis, we performed an explorative stepwise regression analysis considering potential cluster effects. We identified supervisor support for occupational safety and job satisfaction as main factors for our dependent variable company standards. In the previous WorkSafeMed study in two German university hospitals, leadership and job satisfaction were also found as main factors for perceived occupational safety climate among nurses and physicians (48). Thus, our regression analysis indicates that improvements regarding leadership and job satisfaction may have a positive impact on perceived occupational safety climate in general practices, and should be further supported. Predictability, and personal perception of the frequency of occupational risk also remained in the model, but with a smaller influence. The good design of working conditions and the organization of a functioning occupational health and safety structure in the workplace are therefore of central importance. Furthermore, the AVEM dimension subjective importance of work are associated with our dependent variable company standards. In a previous analysis of the baseline data of the IMPROVEjob trial sample, subjective important of work was in the norm (between 4 and 6 points) for the group of practice owner (Mean ± SD = 4.2 ± 1.98) and practice assistants (Mean ± SD = 4.08 ± 2.24), but slightly lower for the group of employed physicians (Mean ± SD = 3.8 ± 2.00) (43). In our opinion, it is not surprising that a subjectively perceived importance of work goes hand in hand with the identification of norms, for example for occupational safety and health, that exist at work. We achieved in our regression analysis all in all a satisfying explained variance of 0.58 *R*^2^ (adjusted for cluster effects). In the WorkSafeMed study, only a low model quality was achieved (48). We therefore assume that our dependent variable company standards served as a better indicator of perceived occupational safety climate than the dependent variable personal perception of the frequency of occupational risks in the former WorkSafeMed study.

The results of our analysis support the need for workplace health management interventions and the promotion of job satisfaction in healthcare settings, and especially in general practices as a common example for micro- and small-sized enterprises. The IMPROVE*job* intervention was one of the first interventions in this area and aimed to reduce psychosocial stressors and promote job satisfaction among practice owners and employees (80). The IMPROVE*job* trial revealed high acceptance among the participants, but showed no significant improvement for job satisfaction (38). This may be due to the fact that the intervention period coincided with the beginning of the COVID-19 pandemic in Germany, as the COVID-19 pandemic with its consequences for the work in general practices had probably a major influence on the effectiveness of the IMPROVE*job* intervention as was stated in Degen et al. (38). Meanwhile, other interventional studies besides the IMPROVE*job* project have focused on mental health and wellbeing in small- and medium-sized enterprises (SMEs). One example is the MENTUPP study (81, 82). The MENTUPP intervention will be conducted in eight European countries and Australia, and targets improvements in mental health and wellbeing for employees in construction, healthcare and information and communication technology sectors (83). Hopefully, further insights regarding the support and improvement of mental health and wellbeing for employees in SMEs will be received, which can also be applied in the healthcare sector.

### Limitations

Several limitations of our study need to be discussed. First, despite very great effort in recruiting general practices for the IMPROVE*job* trial, our study comprised only a small sample of primary care physicians and practice assistants (37). Based on our results and the findings of the other analyses of the data performed so far (37, 39, 41, 43), we assume that mostly high motivated staff participated in the study. Furthermore, we presume that the external validity of our study results is quite limited and we cannot exclude a response bias. Second, we included in this analysis only cross-sectional data from the baseline survey in 2019 and early 2020. So, we cannot draw any conclusions about causality. Regarding the cross-sectional characteristics of the baseline date, attitudes on occupational safety climate can differ during time, and may have changed since our survey due to the COVID-19 pandemic. Another limitation lies in the design of the study itself. In the original IMPROVE*job* trial, an intervention to promote job satisfaction was developed and tested. Job satisfaction was the primary outcome, and occupational safety climate was only regarded as secondary outcome. Thus, the questionnaire used in our study cannot be regarded as a pure questionnaire for capturing occupational safety climate in this setting. Based on experiences from our previous studies on occupational safety climate (48, 49), we captured occupational safety climate quite comprehensively. Yet, with regard to the length of the questionnaire and primary goal of the IMPROVE*job* trial we decided not to integrate some of the variables in our questionnaire proposed for example by Flin in his model for occupational safety culture in healthcare (e.g., unsafe behaviors, worker injuries) (84).

## Conclusion

Overall, practice owner and employees in general practices rated their occupational safety climate positively. Leadership and job satisfaction were identified as main factors in our regression model. This result confirms earlier study findings and the importance of respective workplace health management interventions. The promotion of leadership and job satisfaction should be more supported in general practice teams. More workplace health management interventions focusing on psychological stress and strain like the multimodal participatory IMPROVE*job* intervention are required and can probably contribute to an improvement of occupational safety climate in GP settings in Germany.

## Data Availability

The datasets presented in this article are not readily available because there are no plans to grant access to full protocol, participant-level dataset or statistical code as data contain potentially identifying information. Requests to access the datasets should be directed to Anke Wagner, anke.wagner@med.uni-tuebingen.de.
