# Supplementary material for: Shaping a positive occupational safety climate in general practice teams—findings of the baseline survey of the cluster randomized IMPROVEjob trial
Source: Front Public Health. 2025 Apr 16;13:1477930. doi: 10.3389/fpubh.2025.1477930 (PMC12041036; doi:10.3389/fpubh.2025.1477930)
Supplement: Supplementary file 1 [file Data_Sheet_1.docx]

Supplementary Material

Shaping a positive occupational safety climate in general practice teams – findings of the baseline survey of the cluster randomized IMPROVE*job* trial

Anke Wagner^*^, Esther Rind, Stephanie Burgess, Irina Böckelmann, Beatrice Thielmann, Helen Heinz, Achim Siegel, Verena Schröder, Karl-Heinz Jöckel, Anika Hüsing, Claudia Pieper, Anna-Lisa Eilerts, Tanja Seifried-Dübon, Florian Junne, Brigitte Werners, Annegret Dreher, Lukas Degen, Birgitta M. Weltermann, Monika A. Rieger on behalf of the IMPROVE*job* consortium

*** Correspondence:** Dr. Anke Wagner: anke.wagner@med.uni-tuebingen.de

**Supplementary Material 1: STROBE statement [47]**

STROBE Statement—Checklist of items that should be included in reports of ***cross-sectional studies***

|  | Item No | Recommendation | Page |
| --- | --- | --- | --- |
| **Title and abstract** | 1 | (*a*) Indicate the study’s design with a commonly used term in the title or the abstract | Title |
|  |  | (*b*) Provide in the abstract an informative and balanced summary of what was done and what was found | Abstract |
| Introduction | | |  |
| Background/rationale | 2 | Explain the scientific background and rationale for the investigation being reported | Introduction |
| Objectives | 3 | State specific objectives, including any prespecified hypotheses | Introduction |
| Methods | | |  |
| Study design | 4 | Present key elements of study design early in the paper | Materials and Methods |
| Setting | 5 | Describe the setting, locations, and relevant dates, including periods of recruitment, exposure, follow-up, and data collection | Materials and Methods |
| Participants | 6 | (*a*) Give the eligibility criteria, and the sources and methods of selection of participants | Materials and Methods |
| Variables | 7 | Clearly define all outcomes, exposures, predictors, potential confounders, and effect modifiers. Give diagnostic criteria, if applicable | Materials and Methods |
| Data sources/ measurement | 8* | For each variable of interest, give sources of data and details of methods of assessment (measurement). Describe comparability of assessment methods if there is more than one group | Materials and Methods |
| Bias | 9 | Describe any efforts to address potential sources of bias | - |
| Study size | 10 | Explain how the study size was arrived at | Materials and Methods |
| Quantitative variables | 11 | Explain how quantitative variables were handled in the analyses. If applicable, describe which groupings were chosen and why | - |
| Statistical methods | 12 | (*a*) Describe all statistical methods, including those used to control for confounding | Materials and Methods |
|  |  | (*b*) Describe any methods used to examine subgroups and interactions | - |
|  |  | (*c*) Explain how missing data were addressed | Materials and Methods |
|  |  | (*d*) If applicable, describe analytical methods taking account of sampling strategy | - |
|  |  | (*e*) Describe any sensitivity analyses | - |
| Results | | |  |
| Participants | 13* | (a) Report numbers of individuals at each stage of study—eg numbers potentially eligible, examined for eligibility, confirmed eligible, included in the study, completing follow-up, and analysed | Results |
|  |  | (b) Give reasons for non-participation at each stage | Materials and Methods |
|  |  | (c) Consider use of a flow diagram | - |
| Descriptive data | 14* | (a) Give characteristics of study participants (eg demographic, clinical, social) and information on exposures and potential confounders | Results |
|  |  | (b) Indicate number of participants with missing data for each variable of interest | - |
| Outcome data | 15* | Report numbers of outcome events or summary measures | Results |
| Main results | 16 | (*a*) Give unadjusted estimates and, if applicable, confounder-adjusted estimates and their precision (eg, 95% confidence interval). Make clear which confounders were adjusted for and why they were included | - |
|  |  | (*b*) Report category boundaries when continuous variables were categorized | - |
|  |  | (*c*) If relevant, consider translating estimates of relative risk into absolute risk for a meaningful time period | - |
| Other analyses | 17 | Report other analyses done—eg analyses of subgroups and interactions, and sensitivity analyses | - |
| Discussion | | |  |
| Key results | 18 | Summarise key results with reference to study objectives | Discussion |
| Limitations | 19 | Discuss limitations of the study, taking into account sources of potential bias or imprecision. Discuss both direction and magnitude of any potential bias | Discussion |
| Interpretation | 20 | Give a cautious overall interpretation of results considering objectives, limitations, multiplicity of analyses, results from similar studies, and other relevant evidence | Discussion |
| Generalisability | 21 | Discuss the generalisability (external validity) of the study results | Discussion |
| Other information | | |  |
| Funding | 22 | Give the source of funding and the role of the funders for the present study and, if applicable, for the original study on which the present article is based | Funding |

*Give information separately for exposed and unexposed groups.

**Note:** An Explanation and Elaboration article discusses each checklist item and gives methodological background and published examples of transparent reporting. The STROBE checklist is best used in conjunction with this article (freely available on the Web sites of PLoS Medicine at http://www.plosmedicine.org/, Annals of Internal Medicine at http://www.annals.org/, and Epidemiology at http://www.epidem.com/). Information on the STROBE Initiative is available at www.strobe-statement.org.

**Supplementary Material 2: Comparison of items and scales to assess occupational safety climate available from data collection at baseline and follow-up stratified by intervention and control group and by the three occupational groups**

| **Variables** | **Time of data collection** | **Intervention**  **group** | | | | | **Control**  **group** | | | | |
| --- | --- | --- | --- | --- | --- | --- | --- | --- | --- | --- | --- |
|  |  | **Practice owner**  **n=**  **Mean±SD** | **Employed physicians**  **n=**  **Mean±SD** | | | **Practice assistants**  **n=**  **Mean±SD** | **Practice owner**  **n=**  **Mean±SD** | **Employed physicians**  **n=**  **Mean±SD** | | | **Practice assistants**  **n=**  **Mean±SD** |
| Evaluation of information on perceived hazards and health risks at work^1^ (single item, 1=positive, 5=negative) | Baseline | n=44  2.1±0.9 | | n=10  2.1±0.9 | n=123  2.0±0.9 | | n=37  1.9±1.0 | | n=18  1.9±0.5 | n=121  1.9±0.9 | |
|  | Follow-up | n=29  1.9±0.8 | | n=4  1.8±0.5 | n=78  2.2±1.0 | | n=26  1.8±1.0 | | n=11  2.1±0.8 | n=90  1.9±0.9 | |
| Assessment of specific protective measures related to work-related infectious diseases^2^  (index, 7 items, 1=positive, 5=negative)) | Baseline | n=46  1.7±0.5 | | n=10  1.6±0.5 | n=126  1.7±0.5 | | n=38  1.6±0.5 | | n=18  1.6±0.5 | n=125  1.6±0.5 | |
|  | Follow-up | n=29  1.4±0.4 | | n=4  1.4±0.5 | n=80  1.4±0.4 | | n=27  1.4±0.3 | | n=11  1.7±0.5 | n=92  1.4±0.4 | |
| Personal perception of the frequency of occupational risks^2^  (index, 4 items, 1=negative, 5=positive) | Baseline | n=46  3.9±0.6 | | n=10  4.2±0.8 | n=126  4.0±0.7 | | n=38  3.8±0.8 | | n=18  3.9±0.5 | n=127  4.1±0.7 | |
|  | Follow-up | n=29  3.8±0.7 | | n=4  4.1±0.6 | n=80  3.9±0.7 | | n=27  3.9±0.6 | | n=11  3.9±0.7 | n=92  4.2±0.6 | |
| Supervisor support for occupational safety^2^ (index, 3 items, 1=negative; 5=positive) (only employed  physicians and practice  assistants) | Baseline | - | | n=10  4.1±0.5 | n=126  4.0±0.9 | | - | | n=18  4.2±0.7 | n=127  4.1±0.7 | |
|  | Follow-up | - | | n=4  4.0±0.7 | n=80  3.8±0.9 | | - | | n=11  4.5±0.5 | n=92  4.1±0.7 | |
| Level of knowledge regarding legal occupational health and safety regulations^1^ (index, 6 items, 0=negative, 3=positive)  (practice owners only) | Baseline | n=46  1.7±0.6 | | - | - | | n=38  1.7±0.6 | | - | - | |
|  | Follow-up | n=29  1.8±0.4 | | - | - | | n=27  1.8±0.7 | | - | - | |

SD, standard deviation;

^1^Questions from the Evaluation of the Joint German Occupational Safety and Health Strategy (51).

^2^WorkSafeMed study (48, 49).

**Supplementary Material 3: Single items of the modified version of the scale company standards (Betriebliche Normen [55, 56]) used in the baseline survey of the IMPROVE*job* trial**

| **Single Items in English*** | **Single Items in German** |
| --- | --- |
| Health is a top priority in the practice. | In der Praxis wird Gesundheit großgeschrieben. |
| The practice invests a lot in occupational health and safety. | Die Praxis investiert viel in Arbeits- und Gesundheitsschutz. |
| In my experience, the importance of occupational health and safety is underestimated in the practice. | Die Wichtigkeit von Arbeits- und Gesundheitsschutz wird meiner Erfahrung nach in der Praxis unterschätzt. |
| Managers are not interested in the health of their employees. | Die Führungskräfte interessiert die Gesundheit ihrer Mitarbeiter nicht. |
| Nothing is done in the practice to ensure that employees remain healthy. | In der Praxis wird nichts dafür getan, dass die Mitarbeiter gesund bleiben. |
| The measures to maintain and promote employee health in the workplace are good. | Ich finde die Maßnahmen zur Erhaltung und Förderung der Mitarbeitergesundheit am Arbeitsplatz gut. |
| The practice does not ask whether the working conditions are detrimental to employee health. | In der Praxis wird nicht danach gefragt, ob die Arbeitsbedingungen der Mitarbeitergesundheit schaden. |
| The regulations on occupational health and safety in the practice are good. | Ich finde die Regelungen zum Arbeits- und Gesundheitsschutz in der Praxis gut. |
| What the practice does for health is either insufficient or nonsensical. | Was die Praxis für die Gesundheit tut, ist entweder nicht ausreichend oder unsinnig. |

*translation by the authors

**Supplementary Material 4: Comparison of attitudes towards occupational safety climate stratified by study participants working full- or part-time**

|  | **Full-time workers** | **Part-time workers** | **Significance** | **Effect size** |
| --- | --- | --- | --- | --- |
| **Variables** | **n**  **Mean±SD**  **Median**  **(Min-Max)** | **n**  **Mean±SD**  **Median**  **(Min-Max)** | ***p*** | ***d_Cohen_*** |
| Evaluation of information on perceived hazards and health risks at work^1^  (single item, 1=positive; 5=negative) | n=180  2.0±1.1  2.0  (1-4) | n=166  1.9±1.1  2.0  (1-5) | 0.120^a^ | 0.156 |
| Assessment of specific protective measures related to work-related infectious diseases^2^  (index, 7 items, 1=positive; 5=negative) | n=186  1,7±0.6  1.7  (1-3) | n=170  1.7±0.6  1.6  (1-3) | 0.665^a^ | 0.046 |
| Personal perception of the frequency of occupational risks^2^  (index, 4 items, 5=positive; 1=negative) | n=185  3.8±0.8  4.0  (2-5) | n=172  4.1±0.9  4.3  (2-5) | <0.001^a^ | 0.523 |
| Company standards^3^  (scale, 9 items, 4=positive; 0=negative) | n=177  2.8±0.8  2.9  (0-4) | n=165  2.9±0.9  3.0  (0-4) | 0.088^a^ | 0.185 |
| Occupational safety commitment of the practice^1^  (single item, 1=positive; 4=negative) | n=184  2.3±0.7  2.0  (1-4) | n=169  2.0±0.8  2.0  (1-4) | <0.001^a^ | 0.324 |
| My direct supervisor openly addresses problems concerning occupational safety in our practice^2^  (single item, 5=positive; 1=negative) | n=108  3.6±1.2  4.0  (1-5) | n=162  4.0±1.1  4.0  (1-5) | 0.002^a^ | 0.371 |
| It is important to my direct supervisor that our practice pays great attention to occupational safety^2^  (single item, 5=positive; 1=negative) | n=109  3.6±1.3  4.0  (1-5) | n=161  4.0±1.5  4.0  (1-5) | 0.004^a^ | 0.332 |
| My direct supervisor focuses more on occupational safety than a year ago^2^  (single item, 5=positive; 1=negative) | n=99  2.7±1.1  3.0  (1-5) | n=152  2.7±1.1  3.0  (1-5) | 0.758^a^ | 0.038 |
| Supervisor support for occupational safety^2^  (scale, 3 items, 5=positive; 1=negative) | n=110  3.9±1.0  4.0  (1-5) | n=164  4.1±0.9  4.3  (2-5) | 0.018^a^ | 0.286 |

SD, Standard deviation.

^a^Mann-Whitney-U-test.

^1^Questions from the Evaluation of the Joint German Occupational Safety and Health Strategy (51).

^2^WorkSafeMed study (48, 49).

^3^FAGS questionnaire (Fragebogen zum Arbeits- und Gesundheitsschutz [Questionnaire on Occupational Safety and Health]) (50, 55, 56).
